# Electronic Strategies for Tailored Exercise to Prevent Falls: Evaluating Implementation in Primary Care

**DOI:** 10.1055/a-2896-8054

**Published:** 2026-07-24

**Authors:** Jenna Reisler, Patricia Dykes, Nancy K. Latham, Biai Digbeu, Efstathia Polychronopoulou, Michael Sainlaire, Tien Thai, Mackenzie Kiesman, Erin Hommel

**Affiliations:** 1 12338 The University of Texas Medical Branch at GalvestonGalvestonTexasUnited States; 2 Department of Medicine, General Internal Medicine 370908 Brigham and Women's HospitalBostonMassachusettsUnited States; 3 1861 Brigham and Women's HospitalBostonMassachusettsUnited States; 4 Department of Medicine 370908 Brigham and Women's HospitalBostonMassachusettsUnited States; 5 Division of GeriatricsDepartment of Medicine 12338 The University of Texas Medical Branch at GalvestonGalvestonTexasUnited States

**Keywords:** clinical information systems, clinical decision support, electronic health records, clinical care, ambulatory care, preventive medicine

## Abstract

**Background:**

Falls are the leading cause of injury and injury-related death among older adults. Clinical decision support (CDS) may improve clinician referral and patient access to fall prevention exercise.

**Objectives:**

We evaluated implementation of a CDS intervention linking high-risk older adults to gait and balance exercises and identified patient and clinician factors associated with success.

**Methods:**

Using the Reach, Effectiveness, Adoption, Implementation, and Maintenance (RE-AIM) framework, we evaluated implementation year 1 after a 3-month wash-in period at clinics in an academic health system. Community-dwelling adults aged ≥ 65 seen in person by a physician or advanced practice provider were eligible. We implemented standardized fall risk screening, a CDS alert for positive screens, linked electronic order sets for gait and balance exercise, and preconfigured documentation text. We assessed reach, adoption, implementation, and maintenance; the primary outcome was connection to exercise via order sets or confirmed ongoing gait and balance exercise. Hierarchical logistic regression evaluated associations between patient and clinician characteristics and odds of this outcome.

**Results:**

The CDS alert reached 5,458 of 5,891 eligible patients (92.6%); 1,664 (30.5%) were connected to exercise. Male patient sex (odds ratio [OR]: 0.82; 95% confidence interval [CI]: 0.70–0.95) was associated with lower odds of connection, while multiple recent falls or a fall with injury increased odds (OR: 1.24; 95% CI: 1.02–1.49) compared with self-reported fear of falling. Variation in adoption by clinician was substantial (intraclass correlation coefficient approximately, 30%). No significant differences were found by patient age, race/ethnicity, or insurance, nor for clinician department or qualification.

**Conclusion:**

CDS can identify older adults at high fall risk and prompt referral to gait and balance exercise. However, adoption was modest and varied by clinician, with patient sex and recent fall history influencing uptake. Targeted strategies to improve clinician adoption and address patient biases may enhance implementation.

## Background and Significance

Falls are the leading cause of injury and injury-related death among older adults in the United States, affecting more than 14 million adults aged 65 or older in 2020 and resulting in more than 38,000 deaths in 2021. 1 Most falls do not receive medical attention: only approximately 10% result in injury, and fewer than 20% are reported to primary care physicians (PCP). 2 3 4 Because falls and fall-related injuries are preventable, routine screening and appropriate intervention in primary care are essential.

Clinical practice guidelines emphasize the effectiveness of multicomponent approaches to reduce falls and fall-related injuries. For example, the Centers for Disease Control and Prevention (CDC) Stopping Elderly Accidents, Deaths, and Injuries initiative increases falls screening and reduces fall-related hospitalizations. 5 6 7 8 9 However, widespread implementation is limited by intervention complexity, clinical time constraints, documentation burden, and patient nonadherence. 7 8 9 10 11 Simplifying screening and intervention in primary care may improve adoption and effectiveness of fall reduction programs.

Focusing screening on patients who report recent falls or express fear of falling and prioritizing referrals to exercise interventions is imperative. Prior falls and fear of falling are the most important predictors of future falls, and evidence-based gait and balance exercise is the single most important strategy for fall reduction. 4 12 13 14 15 16 Leveraging advances in computing technology, such as clinical decision support (CDS) and computerized provider order entry (CPOE), could further reduce implementation barriers. 17 18 19

## Objectives

Electronic Strategies for Tailored Exercise to Prevent Falls (eSTEPS) was developed to reduce falls and fall-related injuries among community-dwelling older adults by connecting high-risk patients to exercise interventions via electronic health record (EHR) embedded CDS and CPOE. We utilized an iterative, human-centered design process to develop the CDS and CPOE tools, described in prior publications. 20 21 22 Following fall risk screening, the CDS alert notifies clinicians of positive screens, links to an order set for gait and balance exercise interventions, and populates preconfigured documentation text. 20 21 22 We are conducting a randomized controlled trial (RCT) to evaluate the effectiveness of these interventions in primary care. Here, we present results from a concurrent implementation study assessing the reach, adoption, implementation, and maintenance of the eSTEPS tools in a replication health system.

## Methods

### Setting and Participants

We conducted this study in an academic health system in Southeast Texas comprising one university hospital, three community hospitals, and multiple ambulatory clinics. The Departments of Community Based Clinics (CBC), Family Medicine (FM), Internal Medicine (IM), and Geriatric Medicine (GM) offer primary care services. Targeted clinicians included residents, fellows, faculty physicians, nurse practitioners (NPs), and physician assistants (PAs) within these departments. Eligible patients were community-dwelling adults aged ≥ 65 who completed an in-person visit with a target clinician during the study period; patients in nursing homes or hospice were excluded.

### Intervention Components

Table 1 describes all major intervention components, which were built within the EPIC EHR. At visit onset, rooming staff (medical assistant [MA] or nurse) verbally administered three questions: (1) two or more falls in the past year, (2) any fall with injury in the past year, and (3) fear of falling due to balance or walking problems. While a single noninjurious fall predicts future falls, by focusing on multiple prior falls, any falls with injury, or self-identified gait and balance trouble, we were able to identify those at highest risk of repeated and injurious falls and, therefore, those most likely to benefit from falls reduction interventions. 13 Before eSTEPS, rooming staff performed falls risk screening, but the screen was adapted to the three questions above. We opted against a more comprehensive falls risk assessment given the priority by end users to avoid added burden or disruptions to preexisting workflows. 20 22

**Table 1:** Intervention components

Component	Recipient	Description
Falls risk screening tool Implemented: April 2023	Clinic staff (medical assistant or nurse)	Three-question screen administered at rooming for patients aged ≥ 65 y. Staff records patients' answers to: 1) ≥2 falls within the past year 2) fall with injury within the past year 3) fear of falling due to walking or balance problems. A “yes” to any question indicates a positive screen
EHR CDS Implemented: April 2023 Optimized: May 2023, August 2023, and December 2023	Clinician (MD, DO, NP, PA)	Interruptive CDS triggered by positive screen during office visit upon opening the encounter. Includes: results of fall risk screen, links to best practice guidelines for exercise, prompts to open exercise-oriented order sets, and options to add appropriate fall risk diagnostic codes to the problem list. Clinicians may defer the alert if not addressed in the encounter or inactivate it when exercise interventions are deemed temporarily or permanently inappropriate. Optimized May 2023 and December 2023 to repair firing logic. Optimized August 2023 to add deferral for patient refusal
Structured order set Implemented: April 2023 Optimized: August 2023	Clinician (MD, DO, NP, PA)	Fall prevention exercise recommendations order panel includes options for: formal gym-based physical therapy (PT), home-based PT, patient self-directed home exercise programs Smart text incorporates fall risk plan into clinician's documentation. Optimized August 2023 to preselect documentation smart text and to prepopulate PT orders with indication for gait/balance exercise
Education Implemented: April 2023 Repeated: August 2023	Clinic staff and clinicians	Clinic staff received education about the program through electronic workflow bulletins. Physicians, physician assistants, and nurse practitioners received education through electronic workflow bulletins and in-person or virtual departmental meetings
Data monitoring	Study team	Quarterly review of clinical decision support tool including trigger rates and clinician response

A “yes” to any of the three screening questions constitutes a positive screen and triggers an interruptive CDS for the target clinician. The CDS displays screening results, provides links to gait/balance exercise guidelines, prompts use of the order set, and suggests adding fall risk diagnosis codes to the problem list. The order set includes options for an outpatient or home health physical therapy (PT) referral targeting gait and balance, home gait and balance exercise handouts in English or Spanish (adapted from the Otago Exercise Program 23 ), and brief preconfigured documentation text for the progress note. When the CDS fires, clinicians can: (1) mark patients as already active in gait and balance exercise, (2) connect them to gait and balance exercise through the order set, (3) designate temporary or permanent gait exercise ineligibility, (4) defer the CDS to a later visit, or (5) cancel the CDS (temporarily). While the falls screen is performed at each visit, the CDS is scheduled annually; if canceled, it re-fires when the current encounter is re-opened, and if deferred or temporarily ineligible, it re-fires after 30 days.

In the primary RCT, local clinicians opted to integrate the eSTEPS CDS as a noninterruptive care gap, addressed by the MA during in-person visits. Our institution opted to integrate as an interruptive alert for the target clinician based on cultural role preferences and care gaps governance constraints.

### Implementation Process

Table 1 also outlines the implementation and optimization timeline. The intervention was implemented across all primary care sites on a single go-live date in April 2023. We implemented the program system-wide to expedite rollout, incorporating early lessons from the human-centered development phase, the primary site's RCT, and the first 3 months of local implementation as pilot information to guide optimization.

Since workflow changes for clinic staff (MAs and nurses) were limited to the updated falls risk questionnaire, they were educated about the intervention via electronic workflow bulletins. However, target clinicians received workflow bulletins and attended in-person or virtual departmental meetings. The study team performed quarterly data reviews, implementing tool optimizations as necessary. Logic errors causing CDS misfires were corrected in May and December 2023. In August 2023, the CDS was updated to allow dismissal for patient refusal. Simultaneously, the order sets were optimized by prepopulating PT referral indications to gait and balance impairment and by defaulting the preconfigured text into the clinical note.

### Evaluation Framework and Study Period

Measurement followed the Reach, Effectiveness, Adoption, Implementation, and Maintenance evaluation framework for implementation research. 24 As noted previously, effectiveness is being evaluated in a separate RCT with analysis ongoing. Here, we report on (1) reach—the proportion of eligible patients for whom the tools fired, including patient representativeness, (2) adoption—how clinicians engaged with the interventions, including variation by clinic setting, (3) implementation—fidelity to the intended outcome of connecting patients to gait and balance exercise, defined as identifying patients either already active in gait and balance exercise or connecting patients to evidence-based gait and balance exercise via the order sets, and (4) maintenance—how implementation was sustained over time. The study period was July 2023 to June 2024, following a 3-month wash-in.

We included patients already performing gait and balance exercise into the implementation measure as the CDS promoted identification of these at-risk individuals, who were previously hidden within the system yet receiving the intended intervention. Of note, we were unable to measure what actions clinicians took within the order set due to limitations in EPIC reporting, so we could not discern use of formal PT referrals versus self-directed home exercise. While PT orders and encounters are separately quantifiable, we did not have a mechanism to confirm PT was for gait or balance and, therefore, did not measure them. We also did not have resources to track dose or adherence to formal or informal exercise plans.

### Data Collection and Statistical Analysis

Patient characteristics, clinician characteristics, and encounter-level engagement with the eSTEPS interventions were extracted quarterly from the EHR by a study statistician. Patient characteristics included age, sex, race/ethnicity, insurance, ZIP code, comorbidities, and hospitalization in the preceding year. Zip code was converted to median household income based on 2023 U.S. Census data. 25 Comorbidity burden was assessed using the Charlson Comorbidity Index (CCI), 26 27 a validated measure that assigns weighted scores to chronic conditions to estimate disease burden and mortality risk. In this study, CCI was calculated using age and grouped ICD-10 codes from the EHR; values could range from 0 to 37, with scores above 3 indicating significant risk. Clinician characteristics included department and qualification/level of training (i.e., clinician type).

Intervention use was recorded at each patient's first in-person primary care encounter with a target clinician during the study period, yielding one encounter per patient. Only patients with a positive screen were included. CDS use was analyzed by fall screen responses, distinguishing patients with a recent fall (≥2 falls or any fall with injury in the past year) from those reporting only gait or balance problems. CDS firings and clinician responses were tracked, categorized as canceled/deferred, ineligible, or connected to exercise. Descriptive statistics, including mean with standard deviation and counts with percentages, are reported for the aggregate cohort and by action taken. Response distributions over time are also presented by month.

We used a two-level hierarchical generalized logistic regression model to examine associations between patient and clinician characteristics (chosen a priori) and the odds of being connected to exercise versus cancelling/deferring the alert; ineligible patients were not included in the model to permit model convergence. Level 1 included all patient characteristics except median income as fixed effects; income was removed from the model due to missing data. Level 2 included clinician as a random intercept with a variance component correlation structure to account for patient clustering within a clinician practice. Clinician-associated fixed effects at Level 2 included department and clinician type. To quantify the variance attributable to the clinician, we calculated the intraclass correlation coefficient (ICC) from a null model with clinician as the random effect and compared it with the ICC from the fully adjusted model. Model fit and comparative model performance were assessed using likelihood ratio testing and the Akaike Information Criterion (AIC). AIC assesses model quality and balances fit against complexity; lower values indicate improved model quality. The likelihood ratio test assesses statistically improved fit of the complex model compared with the null model. All analyses were performed using SAS software version 9.4 (Cary, North Carolina, United States).

## Results

### Reach

**Fig. 1:**
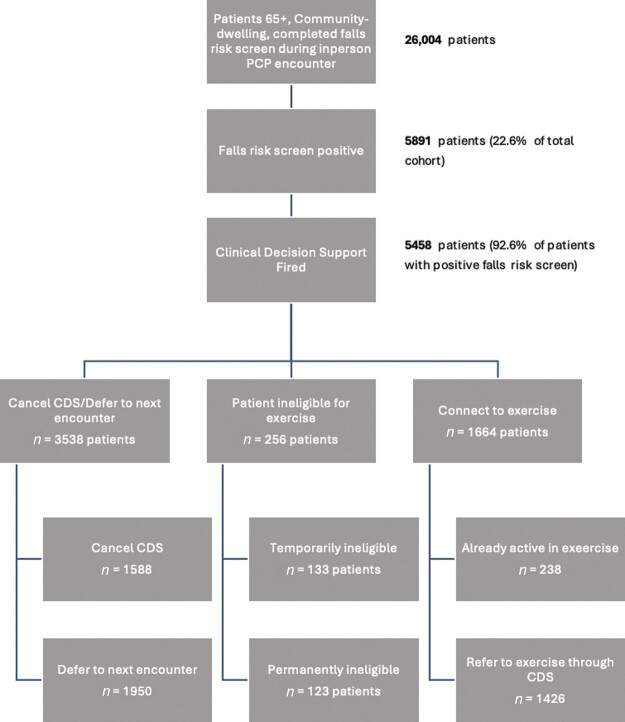
Consort flow diagram of patient identification, screening, and clinical decision support outcomes.

Fig. 1 shows intervention reach. A total of 26,004 community-dwelling older adults completed an in-person primary care encounter with a target clinician during which a falls risk screen was completed. Of these, 5,891 (22.6%) screened positive and were eligible for the intervention.

The CDS fired in 5,458 encounters (92.6% of eligible patients), leaving 433 eligible patients unreached. Of these, 278 had the CDS fire during the wash-in, leaving it inactive at their subsequent encounter. The remaining 155 patients experienced a CDS misfire during a non-PCP encounter, resulting in the CDS being deferred and inactive at their subsequent PCP encounter. This misfire resulted from a builder error during the August 2023 optimization and was corrected in the December 2023 optimization.

Table 2 summarizes characteristics of the 5,458 patients reached by the CDS. The mean age was 76.4 years (SD: 7.7); 64.9% were female, and 67.7% were non-Hispanic White. Most patients were insured under Managed Medicare (59.5%). Median annual income was $77,015. Median income could not be imputed for 183 patients. Patients had a mean CCI of 3.9, and 54.2% had been hospitalized in the past year.

**Table 2:** Characteristics of the cohort by action taken

Characteristic	Total; *n* = 5,458	Ineligible; *n* = 256	Cancel/defer; *n* = 3,538	Connect to exercise; *n* = 1,664
Age, mean (SD)	76.4 (7.7)	76.6 (8.0)	76.3 (7.7)	76.6 (7.8)
Sex, *n* (%)				
Female	3,544 (64.9)	156 (4.4)	2,254 (63.6)	1,134 (32)
Male	1,914 (35.1)	100 (5.2)	1,284 (67.1)	530 (27.7)
Race/ethnicity, *n* (%)				
White	3,657 (67.0)	192 (5.3)	2,344 (64.1)	1,121 (30.7)
Black	794 (14.5)	25 (3.1)	534 (67.3)	235 (29.6)
Hispanic	895 (16.4)	36 (4.0)	574 (64.1)	285 (31.8)
Other	112 (2.1)	3 (2.7)	86 (76.8)	23 (20.5)
Insurance, *n* (%)				
Traditional Medicare	1,931 (35.4)	104 (5.4)	1,255 (65.0)	572 (29.6)
Managed Medicare	3,248 (59.5)	137 (4.2)	2,107 (64.9)	1,004 (30.9)
Other	279 (5.1)	15 (5.4)	176 (63.1)	88 (31.5)
Median income in USD, (IQR) CCI, mean (SD)	77,015 (59,792.0–89,029.0) 3.9 (2.9)	80,199 (63,274–96,284) 3.9 (2.8)	77,015 (63,274–89,029) 3.9 (2.9)	77,015 (59,792–89,029) 3.8 (2.8)
Hospitalization within past 12 mo, *n* (%)				
No Yes	2,500 (45.8) 2,958 (54.2)	116 (4.6) 140 (4.7)	1,606 (64.2) 1,931 (65.3)	778 (31.1) 886 (30.0)
Recent falls, b *n* (%)				
No Yes	1,776 (32.5) 3,682 (67.7)	60 (3.4) 196 (5.3)	1,224 (68.9) 2,314 (62.9)	492 (27.7) 1,172 (31.8)
Reported balance or gait problems, *n* (%)				
No Yes	2,156 (39.5) 3,302 (60.5)	122 (5.7) 134 (4.1)	1,378 (63.9) 2,160 (65.4)	656 (30.4) 1,008 (30.5)
Encounter department, *n* (%)				
CBC	2,813 (51.5)	112 (4.0)	1,907 (67.8)	794 (28.2)
FM	892 (16.3)	45 (5.0)	498 (55.8)	349 (39.1)
IM	888 (16.3)	36 (4.1)	626 (70.5)	226 (25.5)
GM	865 (15.8)	63 (7.3)	507 (58.6)	295 (34.1)
Clinician type, *n* (%)				
Faculty	3,470 (63.6)	156 (4.5)	2,284 (65.8)	1,030 (29.7)
APP	1,414 (25.9)	80 (5.7)	895 (63.3)	439 (31.0)
Resident/fellow	574 (10.5)	20 (3.5)	359 (62.5)	195 (34.0)

Abbreviations: APP, advanced practice provider; CBC, community-based clinics; CCI, Charlson Comorbidity Index; FM, family medicine; GM, geriatric medicine; IM, internal medicine; IQR, interquartile range; SD, standard deviation.

a The census data were missing median income for some zip codes, applying to 183 patients.

b Recent falls = ≥2 falls or 1 fall with injury within the past year.

### Adoption and Implementation

Fig. 1 presents clinician adoption and implementation of interventions. Of the 5,458 patients reached by the CDS, clinicians marked 256 (4.7%) as temporarily or permanently ineligible for exercise. The CDS was canceled or deferred for 3,538 patients (64.8%). The remaining 1,664 patients (30.5%) were connected to exercise, with 238 identified as already active in gait and balance exercise and 1,426 referred through the linked order set.

During the study period, we identified 244 target clinicians across 24 clinics in the four departments. Two clinicians (0.8%) and one clinic (4.2%) had no eligible patient encounters. Table 2 shows the distribution of eligible encounters by department and clinician type. Most encounters occurred in CBC (51.5%), followed by IM and FM (both 16.3%) and GM (15.8%). Faculty physicians conducted 63.6% of visits, APP clinicians 25.9%, and residents/fellows 10.5%. Adoption was highest in FM (39.1% of patients connected to exercise) and lowest in IM (25.5%). By clinician type, residents/fellows had the highest adoption (34%).

Appendix (available in the online version only) illustrates the relationship between clinician encounter volume and the percentage of patients connected to exercise. Encounter volume varied, from 1 to 295 eligible encounters per clinician. The scatter plot demonstrates a weak negative association between encounter volume and connection to exercise ( *R* ^2^ = 0.013, *p* = 0.078), suggesting that higher encounter volume trended toward a lower percentage of connection to exercise. When stratified by encounter volume quartiles, there was no statistically significant trend in performance. Variability remained substantial within each quartile, reflected by wide interquartile ranges.

Table 3 presents results from the two-level hierarchical generalized logistic regression model. In the null model, the ICC for clinicians was 30.7%, indicating that approximately 30% of the variance was attributable to differences in clinician behavior. AIC for the null model was 5,457.9. After adjusting for covariates, clinician ICC decreased to 29.7%, while AIC improved to 5,289.4, suggesting better model quality. The likelihood ratio test was also statistically significant, indicating improved fit of the adjusted model.

**Table 3:** Hierarchical multivariable model, odds of connecting to exercise

Characteristic	Odds Ratio	95% CI
Age	1.00	0.99–1.01
Sex		
Female	—	—
Male	**0.82**	**0.70–0.95**
Race/ethnicity			
White	—	—
Black	1.06	0.86–1.32
Hispanic	1.02	0.84–1.24
Other	**0.57**	**0.33-0.99**
Insurance		
XXX	—	—
XXX	1.07	0.92–1.24
Other	1.22	0.87–1.72
CCI	0.97	0.95–0.99
Recent falls a		
N	—	—
** Y**	**1.24**	**1.02–1.49**
Reported balance or gait problems		
N	—	—
Y	1.19	0.99–1.41
Hospitalization within past 12 months			
N	—	—
Y	0.92	0.80–1.07
Encounter department			
CBC	—	—
FM	1.39	0.81-2.39
IM	0.69	0.39-1.19
GM	0.64	0.27-1.48
Clinician type			
Faculty	—	—
APP	0.86	0.51-1.45
Resident or fellow	1.03	0.63-1.68

Abbreviations: CBC, community based clinics; FM, family medicine; IM, internal medicine; GM, geriatric medicine; APP, advanced practice provider; CI, confidence interval.

a Recent falls = ≥ 2 falls or 1 fall with injury within the past year.

Male patients had lower odds than female patients of being connected to exercise (odds ratio [OR]: 0.82, 95% confidence interval [CI]: 0.70–0.95). Higher CCI was associated with lower odds of connection (OR: 0.97, 95% CI: 0.95–0.99). Patients reporting recent falls had higher odds of connection than those reporting only gait or balance impairments (OR: 1.24, 95% CI: 1.02–1.49). No significant association was found between connection to exercise and race/ethnicity for Black or Hispanic patients compared with non-Hispanic White patients. Patients listed as “other” race/ethnicity had lower odds of connection compared with non-Hispanic White patients (OR: 0.57, 95% CI: 0.330.99); however, the limited sample size and wide confidence interval make the significance of this finding uncertain. No significant associations were found for age, insurance, recent hospitalizations, clinic department, or clinician type.

### Maintenance

**Fig. 2:**
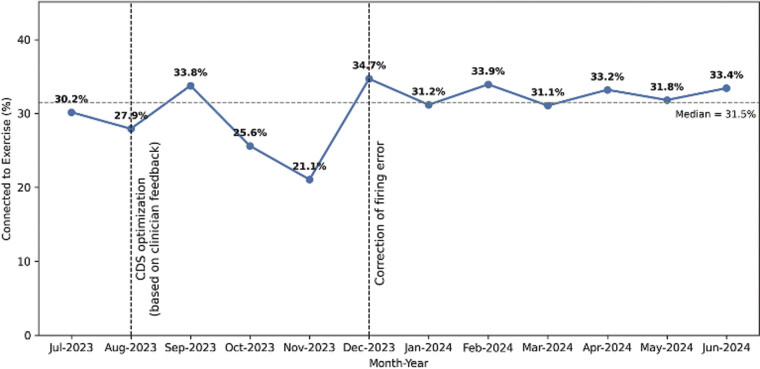
Maintenance of implementation as percent of patients connected to exercise per study month. Note: Vertical dashed lines indicate intervention changes (CDS optimization and firing-logic correction). Percentage reflects the ratio of patients determined to be active in exercise or referred to exercise to the total number of eligible patients.

**Fig. 3:**
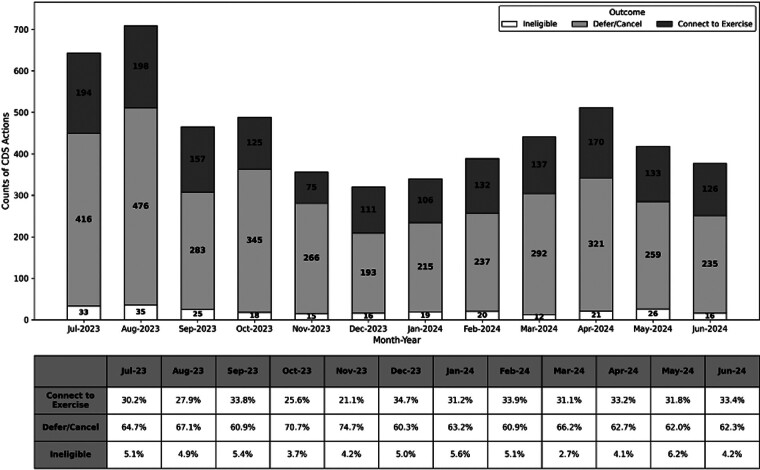
Counts and proportions of CDS responses per month.

Fig. 2 displays the maintenance of implementation during the study period. In July 2023, 30.2% of eligible patients were connected to exercise, declining to a low of 21.1% in December 2023, coinciding with CDS misfires. After CDS correction, connection rates increased and remained above 30% for the remainder of the study period. The proportions of ineligible patients and deferred or canceled alerts remained consistent over time ( Fig. 3 ).

## Discussion

Following human-centered design principles, we successfully implemented eSTEPS within a large academic health system. We achieved broad patient reach, though adoption varied by clinicians regardless of department or clinician type. Overall, 30.5% of eligible patients were identified as connected to exercise or newly referred, with implementation rates remaining stable after tool optimization. In addition to clinician behavior, key patient characteristics influenced odds of connection. Below, we discuss successes and barriers related to patients, clinicians, and system factors, including opportunities to enhance future implementation.

We successfully identified community-dwelling older adults at high fall risk across primary care departments. Patients were routinely screened for fall risk during clinician visits, with more than one in five screening positive, consistent with CDC estimates that one in four older adults report falling annually. 1

Interestingly, a few key patient characteristics influenced the odds of being connected to exercise. Male patients were less likely than female patients to be connected, suggesting potential resistance to the intervention. Men are historically less likely to report falls or discuss fall risk with physicians, 28 and may prioritize fall reduction programs for women while being less receptive themselves. 29 To better reach men, future conversations could frame exercise around maintaining strength and independence while also emphasizing the greater association between falls and mortality in men compared with women. 30

Patients with higher comorbidity burden, measured by the CCI, were also less likely to be connected to exercise. High-risk older adults often have multiple competing health needs, which may lead to clinician override or dismissal of electronic alerts 31 and limit time for preventive counseling. 32 Clinicians may also question exercise feasibility for medically complex patients. 33 Strategies to increase gait and balance exercise referrals in patients with multimorbidity could include optimizing use of and counselling within preventive visits such as the annual Medicare wellness visit. System enhancements prioritizing alerts for medically complex patients could reduce alert fatigue and limit cancellation and deferral rates. 34

Of importance, patients with ≥ 2 falls or at least one fall with injury in the past year were more likely connected to exercise compared with patients only reporting fear of falling. This may reflect clinicians' emphasis on secondary rather than primary falls prevention. Prior literature confirms primary care clinicians often prioritize active disease treatment over prevention due to a combination of training, personal beliefs, and time and resource constraints. 35 Patients and clinicians alike may benefit from education on the predictive performance of patient-reported fear of falling on future falls. More in-depth gait and balance assessment within primary care practices could be targeted to patients who self-identify gait and balance disorders to better define their needs and appropriateness for exercise interventions.

Regarding adoption and implementation, intervention success was primarily driven by individual clinician behavior, separate from department and clinician type. A study by Overhage and colleagues demonstrated that EHR usage patterns by PCP varied at the physician level, much more significantly than the practice or system level. 36 Several factors may contribute to this variability. Differences in familiarity with the intervention, perceived usefulness of the recommendations, or varying comfort discussing may influence engagement. This finding also supports prior evidence that clinician behavior is difficult to change, with time to change and rates of change varying substantially among practices. 37 38 39 Effective techniques to spur change include active rather than passive education, audit and feedback, local champions, and incentives for high performers. 37 38 Future enhancements could incorporate formal audit and feedback cycles with hands-on training targeting high and low adopters to reinforce best practices and address barriers to adoption.

Despite variable clinician performance, the eSTEPS CDS tools achieved and maintained an overall success rate greater than 30%, which is comparable to literature reporting interruptive CDS action rates of 8 to 40%. 31 40 eSTEPS development was iterative, followed human-centered design principles, and was led by a front-line user, which likely contributed to its modest early success. Additional evidence-based strategies can further optimize interruptive alert acceptance, including following the CDS Five Rights framework, using data visualization tools to monitor performance and permit anomaly detection, reviewing data with a multidisciplinary team, and optimizing governance over the approval and maintenance of CDS tools. 40 41

The current eSTEPS tools meet four of the Five Rights parameters. We identified high-risk primary care patients and connected them to evidence-based exercise interventions through a straightforward EHR-embedded alert and streamlined order set. However, results suggest that the interruptive alert is not optimized for the right time in patient care. The CDS fires when the encounter is opened following a positive screening, which may occur during precharting, before shared decision-making can occur, or at visit onset, when other priorities take precedence. As an interruptive CDS, immediate action is required, which likely contributes to frequent cancellation and/or deferral. While early alerts are helpful to draw attention to an underidentified condition and to incorporate counselling into the visit, the EHR functionality should also support delayed action, such as at order entry. Collaborating with EHR vendors to design early alerts which permit delayed action could substantially increase exercise intervention rates.

### Strengths and Limitations

Strengths of this study include the large sample size, comprehensive EHR integration, and hierarchical modeling to assess multi-level associations. However, there were limitations. Conducted at a single academic health system, findings may not be generalizable due to variations in patient demographics, clinic workflows, and EHR systems. Unmeasured factors, including patient volume, unpredictable and competing clinical conditions, and patient/clinician preferences, likely influenced CDS responses. Misclassification is also possible; patients may underreport falls or may report activity in exercise that is not properly targeting gait and balance.

The observational design, lacking a control group or randomization, precludes causal inferences. Additionally, we did not measure exercise type or patients' compliance with the exercise programs to which they were referred. Thus, program success may be more limited than our analysis suggests. Results of the RCT assessing effectiveness will shed more light on the implications of the eSTEPS tools.

## Conclusion

The eSTEPS intervention relied on three integrated EHR tools that ensured consistent identification of patients at high fall risk and streamlined evidence-based gait and balance exercise referrals. After correcting early-firing errors and incorporating PCP feedback, the tools achieved high reach, though adoption was modest. Addressing clinician behavior change and exploring clinician and patient biases related to male sex, multimorbidity, and primary versus secondary fall prevention should enhance implementation. Finally, alert prioritization, frequency, and within-encounter integration represent targets for EHR system optimization. This work underscores the importance of evidence-based processes in optimizing electronic CDS tools and highlights the power of data science in understanding patient and clinician contributions to system-level change.

## Notes

**Clinical Relevance Statement**
Implementation of an electronic health record-embedded clinical decision support tool in primary care successfully identified older adults at high fall risk and facilitated delivery of evidence-based gait and balance exercise interventions. However, variation in clinician adoption and patient characteristics limited uptake, with only 30.5% of high-risk patients connected to exercise. Optimizing these tools to address patient, clinician, and system factors may improve the consistent delivery of fall prevention interventions in routine practice.

## Notes

**Multiple-Choice Questions**

1. Which design feature of the eSTEPS intervention best supports scalability across primary care settings?
  - Dependence on specialized personnel.
  - Manual referral processes.
  - Electronic health record integration.
  - Stand-alone patient education materials.
  **Correct Answer** : The correct answer is option c. Scalability in primary care interventions is enhanced when tools are embedded within widely used systems, such as the EHR. EHR integration allows interventions to be deployed across clinics without requiring additional staff, external platforms, or extensive retraining. The eSTEPS intervention leveraged existing EHR infrastructure to identify patients at risk for falls and facilitate tailored exercise recommendations. This design supports broader dissemination and adaptability across diverse primary care environments while maintaining consistency in delivery.
2. Which outcome is most directly influenced by effective clinical decision support in preventive care?
  - Reduction in hospital length of stay.
  - Increased use of diagnostic testing.
  - Improved delivery of evidence-based interventions.
  - Decreased need for specialist referrals.
  **Correct Answer** : The correct answer is option c. Clinical decision support is designed to improve the delivery of evidence-based interventions by assisting clinicians at the point of care. In preventive care, CDS can help identify at-risk patients, prompt appropriate interventions, and reduce gaps between evidence and practice. Rather than directly affecting downstream outcomes such as hospital length of stay, CDS primarily influences clinician behavior and care processes. These process improvements may subsequently lead to improved patient outcomes over time.
