# Supplementary material for: Electronic Strategies for Tailored Exercise to Prevent Falls: Evaluating Implementation in Primary Care
Source: Appl Clin Inform. 2026 Jul 24;17(3):642–51. doi: 10.1055/a-2896-8054 (PMC13400137; doi:10.1055/a-2896-8054)
Supplement: Supplementary file 1 — Supplementary Material [file 10-1055-a-2896-8054_29241166.pdf]

Appendix Provider-level variation in exercise connection rates by encounter volume

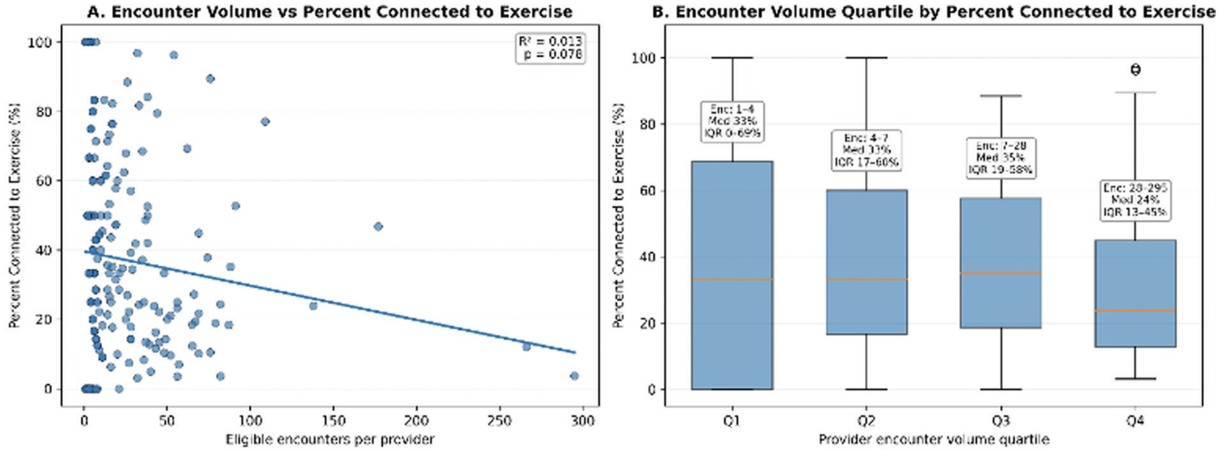

A) Scatter plot showing the relationship between provider encounter volume and percent connected to exercise with fitted linear regression line (R^2 and p-value shown). B) Boxplot of percent of patients connected to exercise stratified by quartiles of provider encounter volume. Boxes represent the interquartile range with median line; whiskers indicate distribution, and points represent outliers. Encounter ranges and corresponding median and interquartile range (IQR) of percent connected to exercise are displayed for each quartile.

© 2026. The Author(s).
